# Developing an Explainable Machine Learning-Based Personalised Dementia Risk Prediction Model: A Transfer Learning Approach With Ensemble Learning Algorithms

**DOI:** 10.3389/fdata.2021.613047

**Published:** 2021-05-26

**Authors:** Samuel O. Danso, Zhanhang Zeng, Graciela Muniz-Terrera, Craig W. Ritchie

**Affiliations:** ^1^Edinburgh Dementia Prevention, Centre for Clinical Brain Sciences, University of Edinburgh Medical School, Edinburgh, United Kingdom; ^2^School of Informatics, University of Edinburgh, Edinburgh, United Kingdom

**Keywords:** early detection, risk factors, Alzheimer's, personalised dementia risk, explainable AI model, ensemble-based learning

## Abstract

Alzheimer's disease (AD) has its onset many decades before dementia develops, and work is ongoing to characterise individuals at risk of decline on the basis of early detection through biomarker and cognitive testing as well as the presence/absence of identified risk factors. Risk prediction models for AD based on various computational approaches, including machine learning, are being developed with promising results. However, these approaches have been criticised as they are unable to generalise due to over-reliance on one data source, poor internal and external validations, and lack of understanding of prediction models, thereby limiting the clinical utility of these prediction models. We propose a framework that employs a transfer-learning paradigm with ensemble learning algorithms to develop explainable personalised risk prediction models for dementia. Our prediction models, known as *source models*, are initially trained and tested using a publicly available dataset (*n* = 84,856, mean age = 69 years) with 14 years of follow-up samples to predict the individual risk of developing dementia. The decision boundaries of the best source model are further updated by using an alternative dataset from a different and much younger population (*n* = 473, mean age = 52 years) to obtain an additional prediction model known as the *target model*. We further apply the SHapely Additive exPlanation (SHAP) algorithm to visualise the risk factors responsible for the prediction at both population and individual levels. The best source model achieves a geometric accuracy of 87%, specificity of 99%, and sensitivity of 76%. In comparison to a baseline model, our target model achieves better performance across several performance metrics, within an increase in geometric accuracy of 16.9%, specificity of 2.7%, and sensitivity of 19.1%, an area under the receiver operating curve (AUROC) of 11% and a transfer learning efficacy rate of 20.6%. The strength of our approach is the large sample size used in training the source model, transferring and applying the “knowledge” to another dataset from a different and undiagnosed population for the early detection and prediction of dementia risk, and the ability to visualise the interaction of the risk factors that drive the prediction. This approach has direct clinical utility.

## Introduction

Dementia is the consequence of a number of progressive neurodegenerative diseases with Alzheimer's disease (AD) accounting for ~60–80% of all types of dementias (Gaugler et al., 2019). AD is considered to be one of the top 10 causes of death, globally. Due to the progressive nature of the disease, people with dementia have different degrees of deterioration in cognition, memory, mental, and other functions (Lyketsos et al., 2002). Moreover, the socioeconomic burden of the disease is estimated to be in the region of one trillion USD per year (World Health Organization, 2017). Dementia has no cure; however, with early detection and diagnosis, it may be possible to delay the onset, which will help reduce the economic burden it currently poses on the society (Prince et al., 2018).

A recent Lancet report has identified modifiable risk factors, which when well-managed could reduce the risk of dementia or delay its onset (Livingston et al., 2020). However, the complexity of the interaction among these risk factors requires computational approaches capable of detecting patterns from these complex interactions to be able to achieve accurate prediction. Meanwhile, machine-learning based approaches have successfully been employed to help identify complex relationships between risk factors and their effect on disease outcomes in various application areas within the care pathway of patients. Examples of such application areas include prediction of pneumonia risk and 30-days readmission in hospital (Caruana et al., 2015), a real-time prediction of patients at the risk of septic shock (Henry et al., 2015), and application of machine learning model in breast screening (Houssami et al., 2017).

Following the above success storeys in the non-dementia domain, numerous attempts are being made to develop machine-learning models for dementia risk prediction. For example, Skolariki et al. (2021) applied machine learning algorithms to predict the likelihood of people with mild cognitive impairment converting to dementia based on features extracted from brain scans. Cui et al. (2019) also applied a recurrent neural network to develop a dementia risk prediction model based on longitudinal features extracted from brain scans. Other studies have also explored features obtained from sources, such as neuropsychological assessments (Barnes et al., 2009; Johnson et al., 2009; Lee et al., 2018; Adam et al., 2020). While these attempts have shown promising results, the prediction algorithms are mostly trained with samples containing diagnosis information and therefore unable to predict beyond the critical window of diagnosis (Prince et al., 2018), making these models ungeneralizable to relatively younger populations (Goerdten et al., 2019). Furthermore, despite these promising results achieved by machine learning-based approaches for dementia, their utility in healthcare settings remains limited partly due to the difficultly in interpreting the outputs of these models (Pellegrini et al., 2018). Interpretable models offer users the confidence and the ability to understand why a certain prediction was made for an individual and the specific underlining factors that led to the prediction. Confidence in how the prediction is made would allow the clinician to communicate this optimally to the patient and intervene. However, lack of confidence on the part of clinicians has resulted in the limited use of powerful machine learning approaches, such as deep learning and ensemble-based learning in developing prediction models for decision support systems in the dementia care pathway. Meanwhile, the complex nature of dementia, which results in complex data structures, makes it imperative to continue to explore these powerful machine learning methods, where traditional approaches, despite their limitations in handling complex data structures (Breiman, 2001), have widely been employed (Goerdten et al., 2019).

We develop and evaluate two ensemble-based interpretable models capable of learning patterns from the complex interactions among risk factors to be able to predict dementia risk at both population and individual levels up to an average of 14 years in advance. Unlike the approaches described above, our final model predicts individual dementia risk based on the parent history of dementia and genetic information about the individual. The prediction models are built using Random Forest (RF) and XGboost algorithms. Briefly, RF like other ensembles of classification and regression trees employs a “divide-and-conquer” strategy in the process of learning by repeatedly partitioning the input data into a number of large classification trees and fitting a prediction model for each tree (Breiman et al., 1984). It then employs the non-parametric bootstrap method (Efron and Tibshirani, 1994) to build a prediction model for each tree. Similarly, the XGBoost also belongs to the family of classification and regression trees and adopts the RF approach to learning. However, XGBoost employs a step-wise, additive approach to sequentially build a prediction model for each tree, while taking into account the difficulties encountered in fitting previous models (Natekin and Knoll, 2013). It is worth noting that RF and XGboost both combine the predictions from weak learners to produce a final model—a process known as “voting.” These algorithms have been demonstrated to be powerful when applied to various problems, such as risk prediction of hypoxaemia during general anaesthesia and surgery (Lundberg et al., 2018).

We argue that our proposed approach provides useful and actionable information to assist clinicians and other users in their decision-making process around diagnosis, prognosis, and management. We also believe that this is an important step for machine learning in neurodegenerative disease research and translation to clinical care. Our approach not only significantly improves the ability for the early detection of neurodegenerative disease but also the ability to explain the predictions from accurate and complex models in order to understand drivers of the prediction for important intervention strategies to be developed.

## Methods

### Overview of the Research Framework

**Figure 1:**
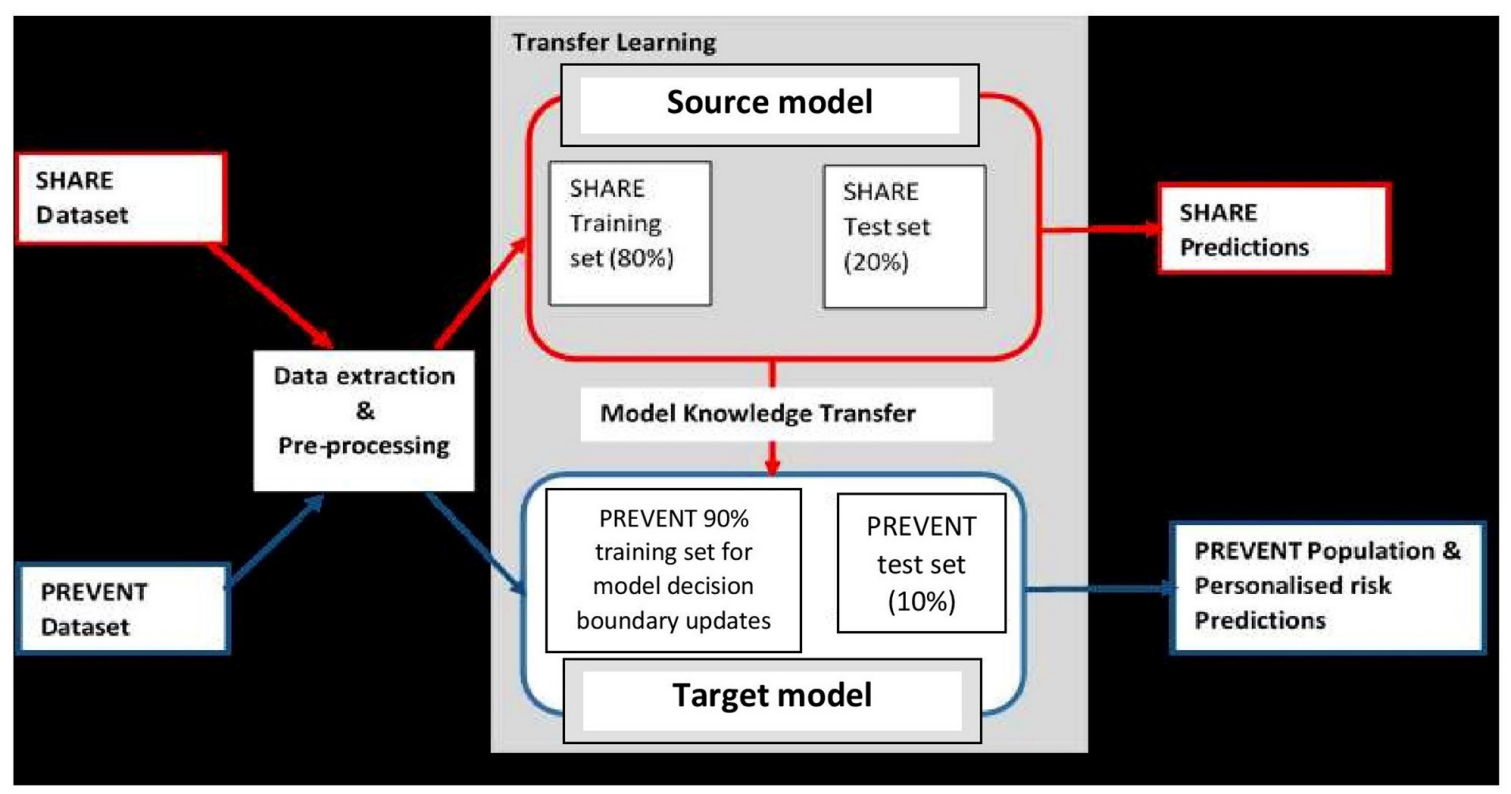
Transfer learning process showing how data extraction and pre-processing procedures are applied to SHARE and PREVENT datasets. A prediction model (Source model) is built using the SHARE dataset with 80% of the data used for training and 20% held-out for testing for SHARE predictions. The Source model is updated with 90% PREVENT training and the updated prediction model (Target model) is applied to PREVENT 10% test set held-out for population as well as personalised risk prediction of dementia.

It is believed that dementia clinically manifests after decades of exposure to risk factors (Ritchie and Ritchie, 2012). Therefore, the aim of this project was to develop a machine learning model capable of predicting the risk of developing dementia decades prior to the onset of the dementia syndrome. To achieve this, the task was formulated as a transfer learning classification problem (Pan and Yang, 2009). This made it possible to develop the machine learning prediction model using the data drawn from different populations and applied the model to another population. Figure 1 illustrates the methodology employed. As the figure shows, unlike traditional machine learning where a model is developed and applied to predict data from the same population, our model was developed using external data source and transferred the knowledge learned from the external population and applied it to data from population of different characteristics. The characteristics of the data sources are discussed in the next section.

### Data Description and Preprocessing

The data sources used in developing the models were obtained from the Survey of Health, Ageing, and Retirement in Europe (SHARE) study (Börsch-Supan et al., 2013) and the PREVENT Dementia programme (Ritchie and Ritchie, 2012). While both SHARE and PREVENT projects are related to dementia research, the rationale and aims of each of the studies vary resulting in differences in the datasets. Table 1 shows a brief description of the datasets. While SHARE population covers 20 European countries with the mean age of 69 years, the PREVENT data, on the other hand, is a relatively younger cohort with the mean age of 52 years drawn from a population limited to the United Kingdom. Further, the SHARE cohort includes individuals with some having been diagnosed with dementia, while the PREVENT cohort contains healthy individuals without a diagnosis of dementia. However, the PREVENT study participants are children of individuals with or without a diagnosed dementia. The study also collects information about the apolipoprotein E (ApoE) genotype of each individual.

**Table 1:** Characteristics of SHARE and PREVENT datasets.

**Data description**	**SHARE data**	**PREVENT data**
Population	20 European countries	The United Kingdom
Number of samples	84,856	473
Mean age	69	52
Number of years of follow-ups	14 years (2004–2015), 2 years interval on average	Only used baseline data
Class distribution	Diagnosis • Diagnosis of Alzheimer's disease—“AD” (*n* = 4,157) • No diagnosis of-Alzheimer's disease diagnosis—“non-AD” (*n* = 80,699)	Parental diagnosis of AD and Apolipoprotein E4 allele (ApoE4) genotype status of individual • Parental diagnosis of AD + ApoE4 status—“High Risk” (*n* = 109) • No parental diagnosis of AD + No ApoE4 status of individual—“Low Risk” (*n* = 364)

Even though both SHARE and PREVENT research programmes have different research aims and objectives, there was a high degree of overlap between the two datasets in terms of data collection. In order to make transfer learning possible, it was important to focus on common data items between the two datasets. Table 2 shows the categories of common variables found in both datasets. We extracted data records from the SHARE dataset and merged the data of individuals across waves 1–6 which covers the period between 2004 and 2015. Therefore, from the SHARE cohort, it was possible to build a prediction model using a longitudinal dataset of 14 years of follow-up data. The PREVENT dataset on the other hand is the baseline data collected between February 2014 and October 2018.

**Table 2:** The common data items between SHARE and PREVENT datasets used to develop the prediction models.

**Data category**	**Data items**
Sociodemographic	• Gender • Age • Education level • Marital status • Had children? • BMI
Self-reported medical history	• Heart attack • Hypertension (high blood pressure) • High cholesterol • Diabetes • Lung disease • Peptic ulcer disease • Parkinson's disease • Emotional disorders • Osteoarthritis
Life style	• Daily activity • Smoking

The difference in data collection protocols used by the studies resulted in structural differences in data. To address these differences, we devised a pre-processing procedure to harmonise the representation of the data items, which were employed as features to train the learning algorithms. All medical history variables were processed to have binary feature representation based on the responses as either condition being present or not present, with a feature value of “1” and “0,” respectively. The Body Mass Index (BMI) as per WHO classification was applied to obtain the following four categories: underweight (<18.5 kg/m^2^), normal (18.5–24.9 kg/m^2^), overweight (25–29.9 kg/m^2^), and obese (>30 kg/m^2^) with feature values of “0,” “1,” “2,” and “3,” respectively Furthermore, “marital status” had categorical entries (“divorced,” “married,” “living with spouses,” “married,” “not living with spouse,” “never married,” and “registered partnership”), and each of these was separately represented as binary based on the response as either “yes” or “no,” with a feature value of “1” and “0,” respectively. The International Standard Classification of Education scheme was applied to “education level” variable to have seven categories with feature value representations (0 = none; 1 = first stage of basic education; 2 = lower secondary education or second stage of basic education; 3 = upper secondary education; 4 = post-secondary non-tertiary education; 5 = first stage of tertiary education; and 6 = second stage of tertiary education). The “daily activity” variables had two categories: “vigorous” and “moderate” sports with each having feature value representations (0 = hardly ever or never; 1 = one to three times a month; 2 = once a week; and 3 = more than once a week). We believe that this method of representation provides information on the activity as well as the intensity of the activity, which can be useful for the learning algorithms. The “smoking” variable was also processed to have a binary representation based on the responses with feature values (0 = never smoked and 1 = current or past smoker). Finally, the SHARE dataset contained data on whether a participant had been diagnosed with Alzheimer's disease (AD) and those without a diagnosis. This was therefore used as the class variable for the prediction model feature values representation (Non-AD = no diagnosis; AD = diagnosis of Alzheimer's dementia). However, in the absence of a diagnosis in the PREVENT dataset, and to facilitate the evaluation of our approach, we employed a classification scheme proposed by Ritchie and Ritchie (2012) to group the participants according to parental clinical status and ApoE genotype. Therefore, participants with a parental dementia diagnosis and ApoE 4 genotype were allocated to a “High-Risk” (HR) group as these individuals were considered to be at high risk of dementia. All other participants were allocated to a “Low-Risk” (LR) group. The final distribution of classes is as follows: SHARE dataset, Non-AD (95%) and AD (5%); PREVENT dataset HR (23%) and LR (77%).

### Building the Prediction Model

We built four ensemble-based prediction models by training RF and XGBoost algorithms. The algorithms were trained by applying a hybrid approach that combines cross-validation and hold out, through a procedure we refer to as *cross-validation with hold out* (Pedregosa et al., 2011). This procedure involved splitting the SHARE data into training and test sets. The training set (D_train), which constituted 80% of the SHARE data, was used to train the algorithms including hyperparameters tuning. The 20% test set (D_eval) was held and used only for the model performance evaluation. Similarly, the PREVENT data was also split into 80% training set (PREV_train) and 20% test set (PREV_eval). The splits were stratified in order to ensure the equal proportion of class representation in both training and test sets. A summary of our cross-validation with hold out training of algorithms procedure is as follows:

- Step 1: We employed a 5-fold cross-validation during training, which randomly split the 80% training set into 5-folds each containing a subset of training (D_train_1−5_) and validation (D_val_1−5_) sets.
- Step 2: We applied a set of initial hyperparameters to train the algorithm to obtain five different models using D_train_1−5_ and D_val_1−5_, to obtain a number of potential hyperparameters from each cross-validation.
- Step 3: We then applied the random search optimization algorithm (Bergstra and Bengio, 2012), to search and choose from a set of potential number of hyperparameters derived from Step 2 to obtain the optimal set of hyperparameters based on the evaluation function of the optimization algorithm. Table 3 shows the set of initial and optimal hyperparameter settings obtained.
- Step 4: Once the optimal hyperparameters are obtained, we then retrained the algorithm using the optimum hyperparameters on the entire training set, D_train.
- Step 5: We applied the procedures in Steps 2–4 for RF and XGBoost to obtain SHARE_RF_pred and SHARE_XGBoost_pred prediction models, respectively.
- Step 6: We evaluated the performance of the prediction models obtained in Step 5 by applying SHARE_XGBoost_pred and SHARE_RF_pred to the hold-out test set (D_eval).
- Step 7: We employed the method proposed by DeLong et al. (1988) to carry out a pairwise comparison of the receiver operating curve (ROC) to compare the performance difference between SHARE_XGBoost_pred and SHARE_RF_pred to determine the best model.
- Step 8: We randomly spit the PREVENT data into 80% training set (PREV_train) and 20% held out test set (PREV_eval). Again, the split was stratified in order to ensure an equal proportion of class representation in both the training and test sets.
- Step 9: We employed a parameter-transfer learning approach as described by Yao and Doretto (2010) to build a target model. This approach assumes that the target shares parameters with the best source model as determined in Step 7. The parameters of the best source model are further updated using the PREV train set. This process adjusted the decision boundaries of the source model to produce PREVENT_target prediction model.
- Step 10: We evaluated the performance of prediction models obtained in Step 9 by applying them to the hold-out test set (PREV_eval).
- Step 11: We trained the XGBoost algorithm using PREV_train and applied procedures into Steps 2–4 to obtain a prediction model (PREVENT_only).
- Step 12: We evaluated the performances of PREVENT_target and PREVENT_only by applying them to the hold-out test set (PREV_eval).
- Step 13: We finally applied the procedures in Step 7 to compare the performance difference between the PREVENT_target and PREVENT_only to determine the best model.

**Table 3:** Hyperparameter settings for prediction models.

**Algorithm**	**Initial parameters**	**Optimal hyperparameter settings**
Random Forest	n_estimators = range (5, 40), max_features = ['auto', 'sqrt', 'log2'], max_depth = range (10, 25), criterion = [gini, entropy]	Bootstrap = True; ccp_alpha = 0.0; class_weight = None; criterion = entropy; max_depth = 24; max_features = sqrt; max_leaf_nodes = None; max_samples = None; min_impurity_decrease = 0.0; min_impurity_split = None; min_samples_leaf = 1; min_samples_split = 2; min_weight_fraction_leaf = 0.0; n_estimators = 33, n_jobs = None; oob_score = False; random_state = None; verbose = 0; warm_start = False
XGBoost	n_estimators = range (1, 20), max_depth = range (10, 25), learning_rate = [.1,.2,.4,.45,.5,.55,.6], colsample_bytree': [.6,.7,.8,.9, 1], booster = gbtree, min_child_weight = [0.001, 0.003, 0.01]	Objective = multi:softprob; base_score = 0.5; booster = gbtree; colsample_bylevel = 1; colsample_bynode = 1; colsample_bytree = 0.7; gamma = 0; gpu_id = −1; importance_type = gain; interaction_constraints = None; learning_rate = 0.5, max_delta_step = 0; max_depth = 24; min_child_weight = 0.003; missing = nan; monotone_constraints = None; n_estimators = 16; n_jobs = 0; num_parallel_tree = 1; random_state = 0; reg_alpha = 0; reg_lambda = 1; scale_pos_weight = None; subsample = 1; tree_method = None; validate_parameters = False; verbosity = None; num_class = 2

### Performance Evaluation

We employed a series of metrics to evaluate the performance of the models based on the D_eval and PREV_eval unseen datasets. As already pointed out, D_eval contained “AD” and “No-AD” which served as the ground truth for the evaluation of SHARE_RF_pred and SHARE_XGBoost_pred models. PREV_eval on the other contained “HR” and “LR” as explained above, and this served as the ground truth for the evaluation of our PREVENT_target and PREVENT_only models. These metrics were primarily based on the following information obtained from the outputs of the prediction models: Refer False Positive (FP), False Negative (FN), True Positive (TP), and True Negative (TN) (Pollack, 1970) for details of these metrics. The comparison of the models was based on geometric accuracy (GA) as expressed in Equation (3) which is derived from Equations (1) and (2) which represent sensitivity and specificity, respectively. GA accounts for both majority and minority class error rates which makes it ideal for imbalanced problems (Kim et al., 2015).

(1) $$\begin{array}{r} Sensitivity=\frac{Number\;of\;TP}{Number\;of\;TP+Number\;of\;FN} \end{array}$$

(2) $$\begin{array}{r} Specificity=\frac{Number\;of\;TN}{Number\;of\;TN+Number\;of\;FP} \end{array}$$

(3) $$\begin{array}{r} Geometric\;Accuracy=\sqrt{\left(Sensitivity*Specificity\right)} \end{array}$$

We also employed area under the receiver operating curve (AUROC) to further explore the robustness of our models, given the wide usage of this metric in medical applications (Mandrekar, 2010). Also, as already stated, a significant test was used to examine the performance differences between the prediction models.

Finally, we employed a method proposed by Taylor and Stone (2009) to examine the efficacy of our transfer learning approach based on a learning ratio as expressed in Equation (4).

(4) $$\begin{array}{r} ratio=\frac{area\;under\;curve\;with\;transfer-area\;under\;curve\;without\;transfer}{area\;under\;curve\;with\;transfer} \end{array}$$

### Feature Importance and Model Interpretability

An important advantage of tree-based algorithms is their ability to provide information on the decisions made around predictions. This information is provided in the form of weights that are assigned to the features as a result of the learning process. The value of weight assigned to a given feature is an indicator of the importance of that feature as determined by the prediction model, which enabled us to examine how each feature was ranked by the prediction models.

We further applied the SHapley Additive exPlanation (SHAP) algorithm to explore the interactions between the features (Lundberg et al., 2018). Briefly, the algorithm is inspired by game theory, where the interaction between features is considered as a “team” of features, with each feature being a member of the team responsible for driving the overall risk. An instance of the interaction between the features registers a set of predicted values produced by the prediction model. These values serve as input for the SHAP algorithm to generate another set of values known as “impact values.” The SHAP values provide a dynamic view of the effects of the interaction between the features to determine the probability of risk and the role of each feature on the individual level. Furthermore, the SHAP algorithm offers the possibility to compare an individual predicted risk probability with a baseline prediction, which is the average predicted probability known as the “base value.”

## Results

### Model Performance Analyses

**Figure 2:**
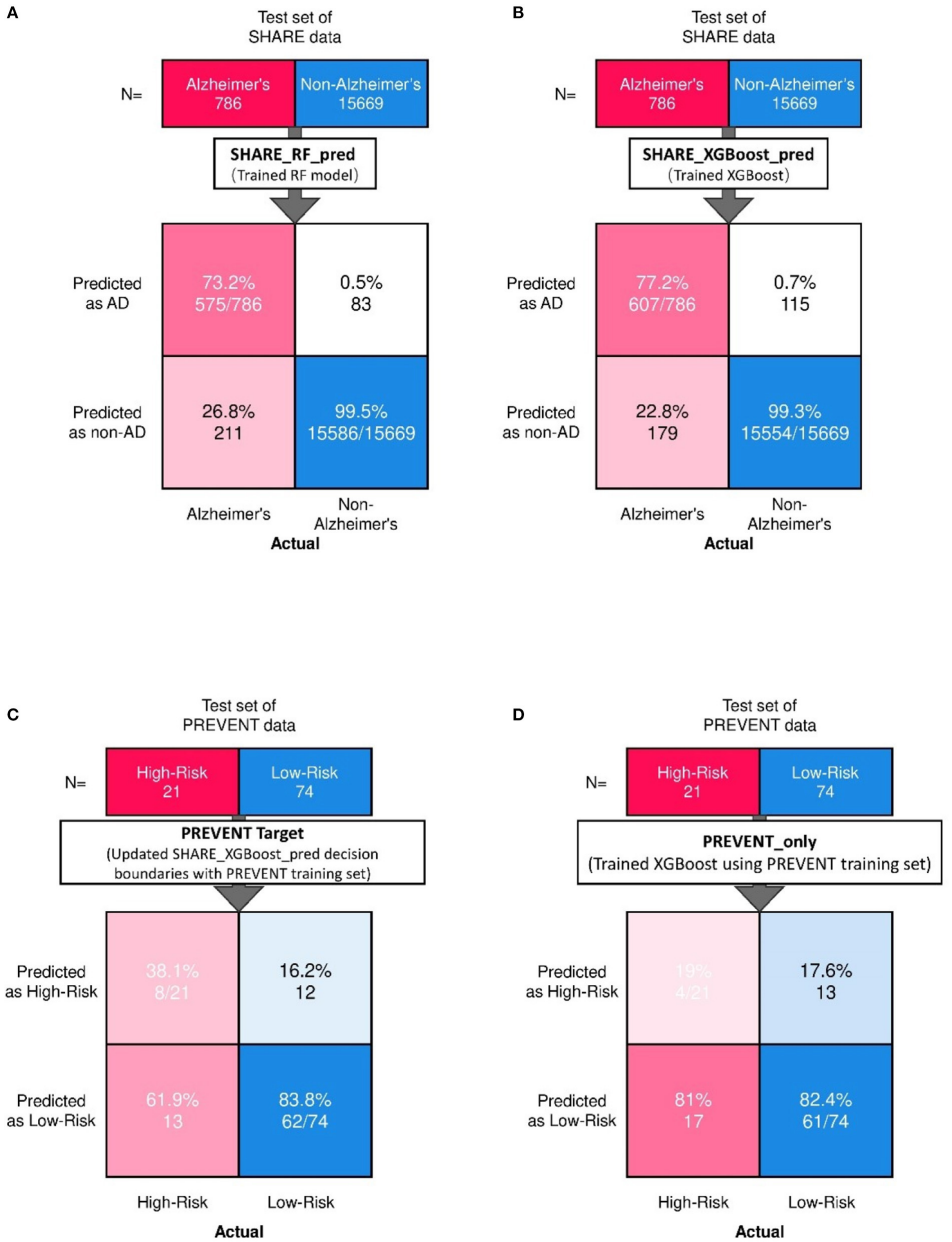
Confusion matrix showing the prediction results from unseen 20% of SHARE test data as predicted by **(A)** Random Forest **(A,B)** XGBoost models. Also showing are the prediction results from 20% unseen PREVENT test data as predicted by **(C)** Updated SHARE_XGBoost_pred decision boundaries with PREVENT training set and **(D)** Trained XGBoost using PREVENT training set.

**Figure 3:**
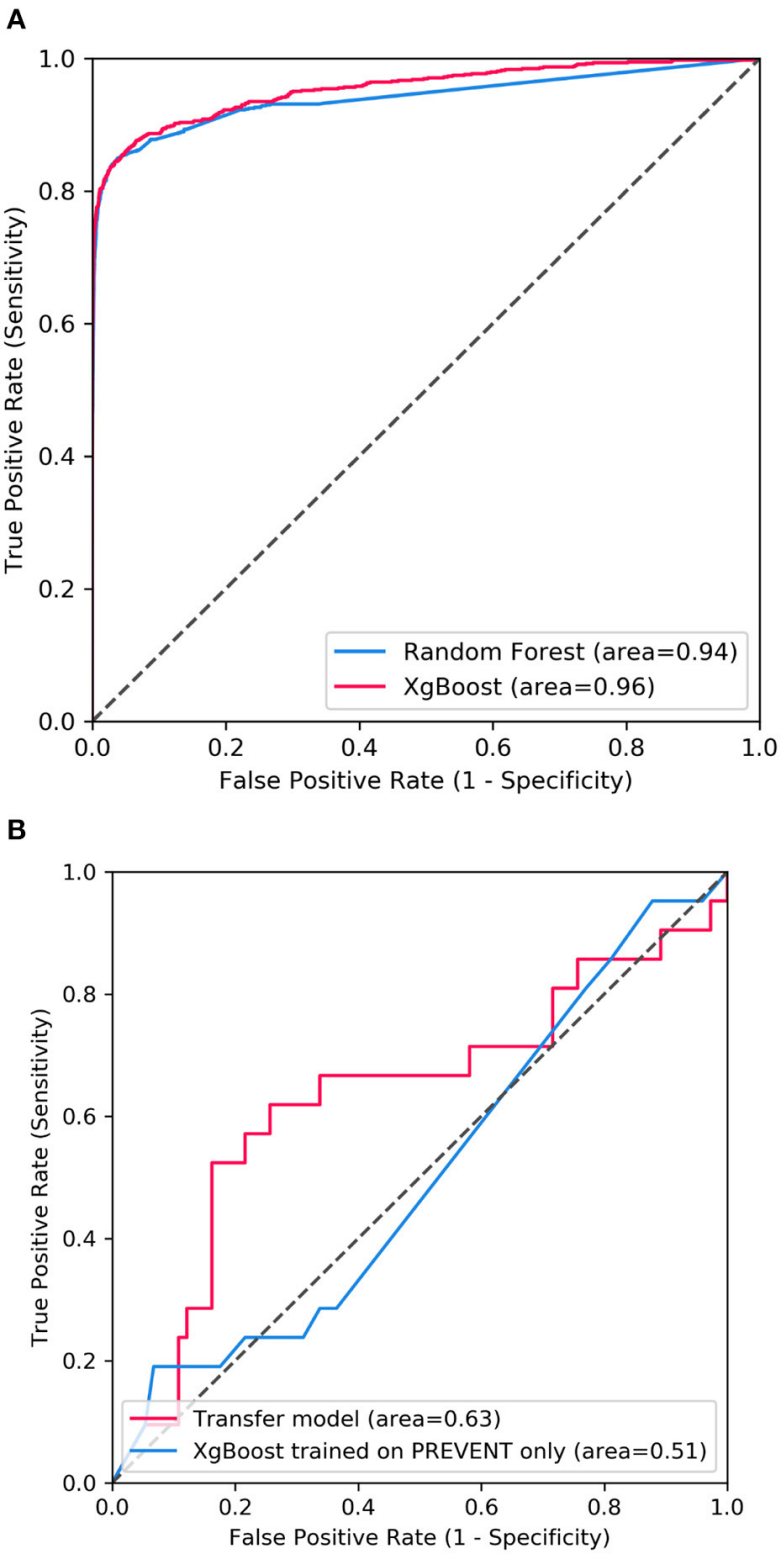
Showing ROC curves with AUROC scores of **(A)** the performance difference between Random Forest and XGBoost prediction models when applied to 20% SHARE unseen test set and **(B)** the performance compassion between XGBoost model updated with PREVENT training set (Transfer model) and XGBoost trained with PREVENT only (PREVENT only model) and applied to 20% PREVENT unseen test set.

Figure 2 shows the confusion matrix of the results obtained when SHARE_RF_pred (Figure 2A) and SHARE_XGBoost_pred (Figure 2B) models were applied to 20% of SHARE unseen test set. The figure also shows the results when PREVENT_target (Figure 2C) and PREVENT_only (Figure 2D) models were applied to 20% of PREVENT unseen test set. Table 4 further shows a summary of the performances obtained. As seen from the table, SHARE_XGBoost achieves a GA of 87%, specificity of 99%, sensitivity of 76%, and AUROC of 96%. In comparison, SHARE_RF_pred achieves a GA of 85%, specificity of 99%, sensitivity of 73%, and AUROC of 94%. Figure 3A shows an AUROC curve comparison between SHARE_RF_pred and SHARE_XGBoost, with SHARE_XGBoost showing a marginal difference in the performance between the two models. A pairwise comparison of the AUROC scores between the two prediction models demonstrates a significant difference in performance (*P* < 0.0001, 95% Confidence Interval: 0.01–0.02), suggesting SHARE_XGBoost as the best performing model.

**Table 4:** Summary of prediction models on the unseen test set.

**Model**	**Sensitivity (%)**	**Specificity (%)**	**Geometric Accuracy (%)**	**AUROC (%)**	***P*-value**	**Transfer learning efficacy (%)**
SHARE_RF_pred	73	99	85	94	*P* < 0.0001	N/A
SHARE_XGBoost_pred	^*^76 (+3%)	^*^99 (0%)	^*^87 (2%)	^*^96 (2%)		
PREVENT_target	^**^38.1 (+19.1%)	^**^84.7 (+2.7%)	^**^56.5 (+16.9%)	^**^63 (+11%)	*P* = 0.2166	20.6%
PREVENT_only	19.0	82.0	39.6	51		

* *Performance comparison in relation to SHARE_RF_pred*.

** *Performance comparison in relation to PREVENT_only*.

Again, as seen from Table 4, PREVENT_target achieves a GA of 56.5%, specificity of 84.7%, sensitivity of 38.1%, and AUROC of 63%. In comparison, PREVENT_only achieves a GA of 39.6%, specificity of 82.0%, sensitivity of 19%, and AUROC of 51%. Figure 3B shows an AUROC curve comparison between PREVENT_target and PREVENT_only, with PREVENT_target showing a marginal difference in performance between the two models. Even though a pairwise comparison of the AUROC scores between PREVENT_target and PREVENT_only, no significant difference in performance is observed (*P* = 0.2166, 95% Confidence Interval: 0.07–0.325), the PREVENT_target model outperformed PREVENT_only model across all the performance metrics as shown in Table 4. There is an increase in the sensitivity of 19.1%, specificity of 2.7%, GA of 16.9%, AUROC of 11%, and a transfer-learning rate of 20.6%.

### Feature Importance Analysis and Interpretability of Personalised Risk Prediction

**Figure 4:**
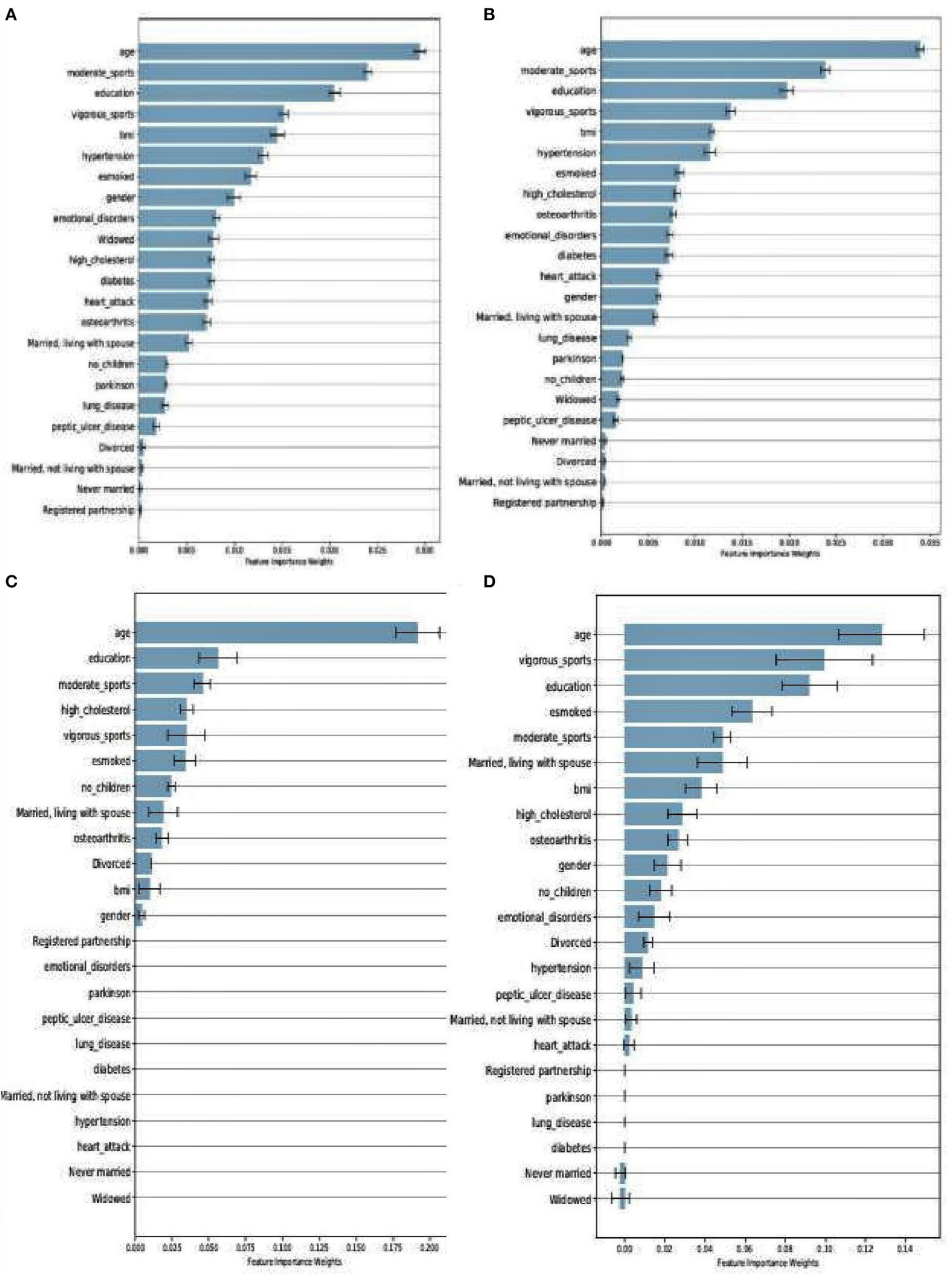
Feature importance as ranked by the weights derived from SHARE_RF_pred **(A)** and SHARE_XGBoost_pred **(B)** prediction models that were trained using SHARE dataset. It also shows the ranking of features of PREVENT only **(C)** and PREVENT target **(D)**.

Even though RF and XGboost are both considered ensemble-based algorithms, the learning strategy tends to differ as briefly discussed. From that score, we examine how both models assessed the importance of the features used in training the models. Figures 4A,B depict a comparison between SHARE_RF_pred and SHARE_XGBoost_pred prediction models on how features were ranked based on the weights assigned. As shown by Figures 4A,B, while significant similarities in the ranking of the features exist between the two models, some striking differences can also be observed. For example, the ranking of the top seven features of both RF and XGBoost appear to be in the same order, with '“age” being the most important feature followed by “moderate sport,” “education,” “vigorous sports,” “BMI,” “hypertension,” and “esmoked.” Some differences in rankings were observed. Where RF ranks “gender” and “emotional disorders” as the 8th and 9th most important features, XGBoost ranks “high cholesterol” and “osteoarthritis,” respectively. Additionally, RF ranks “widowed” as the 10th most important feature, whereas XGBoost ranks “diabetes” as the 10th most important feature, and ranks “widowed” as one of the least important features (ranked 18th).

Similarly, a comparison between PREVENT_only and PREVENT_target shows how these prediction models ranked the features as shown in Figures 4C,D, respectively. Again, while there appear to be some overlaps in the order of feature rankings between the models, some differences can also be observed. For example, “age” remains the most important feature among the two models. A close examination of the top 10 features of the models show some differences in the order of rankings. For example, while PREVENT_only ranks “divorced,” and “no_children” among the top 10, PREVENT_target also ranks “BMI” and “gender” among the top 10, but ranks “divorced,” and “no_children” in the 11th and 13th positions, respectively. Even though these differences in feature rankings can be observed between these two models, the difference is not statistically significant. However, because our PREVENT_target demonstrated some marginal increase in the performance over PREVENT_only, our analysis will be based on the output of PREVENT_target model. A further comparison of the order of rankings of features between SHARE_XGBoost_pred as the source model and our PREVENT_target as the target model also shows 70% overlap among the top 10 features as ranked by both the models. The differences observed include: “emotional_disorders,” “hypertension,” and “diabetes” ranked among the top 10 by SHARE_XGBoost_pred, but ranked by PREVENT_target model at 12th, 14th, and 21st positions, respectively.

**Figure 5:**
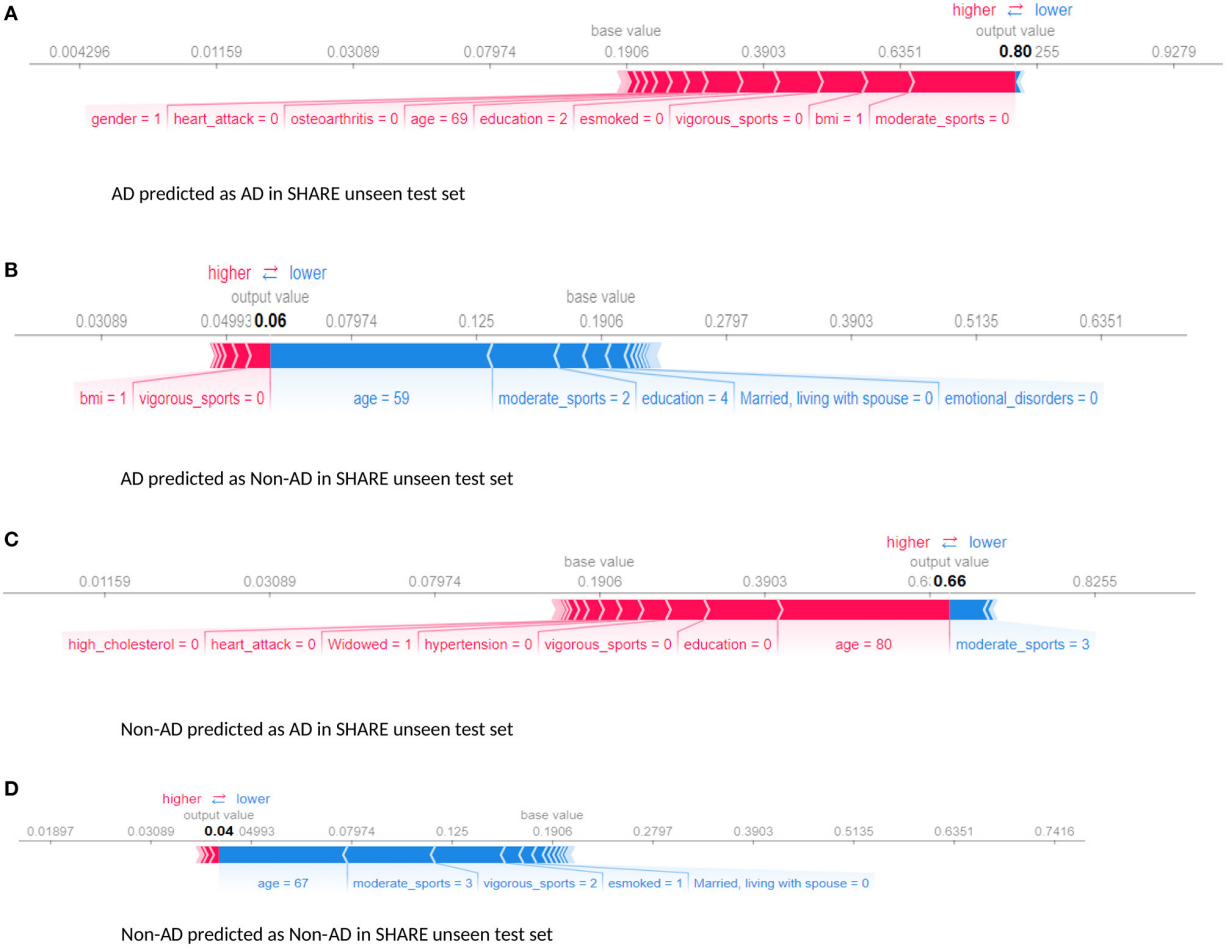
Force plot showing the effect of SHAP values on the interaction of features and the overall prediction at the individual level. This shows examples of prediction outputs with taken from SHARE as predicted by the SHARE model. Features in red show risk factors pushing up the overall probability while those in blue are protective factors pushing down the probability. **(A)** Shows SHARE participants predicted to have AD with 85% probability. **(B)** Shows SHARE participant diagnosed to have AD but has been predicted by the model to be Non-AD with 6% probability. **(C)** Shows a Non-AD participant predicted as AD with 63% probability. **(D)** Shows as Non-AD participant predicted as Non-AD with 4% probability. Feature labels are: **esmoked** (0 = never smoked); **emotional disorders** (0 = no); **hypertension** (0 = no); **osteroarthritis** (1 = yes); **high cholesterol** (0 = no); **heart attack** (0 = no); **education** (2 = lower secondary education or second stage of basic education; 3 = upper secondary education); **moderate sports** (0 = hardly ever, or never, 1 = one to three times a month); **vigorous sports** (0 = hardly ever, or never, 1 = one to three times a month); **no children** (0 = no children); **widowed** (1 = yes); **BMI** [1 = under weight (<18.5)]; **married, living with spouse** (0 = no) and **gender** (1 = male).

Furthermore, we examined the performance of the models at individual levels. Figure 5 shows the visualisation of SHAP values of four randomly selected prediction outputs when SHARE_XGBoost_pred was applied to SHARE unseen test set. Figure 5A shows an individual with AD and correctly predicted by the model, with the probability of 80%. Figure 5B shows an individual with AD which is incorrectly predicted as a non-AD with the probability of 6%. Figure 5C shows an individual without AD predicted as AD with the probability of 66%. Figure 5D also shows an individual without AD and correctly predicted as a Non-AD with the probability of 4%. The figures also show the risk factors that drive each of the probabilities, with red indicating risk factors and blue suggesting protective factors. For example, Figure 5A shows a 69-year-old woman correctly predicted to be living with AD with the probability of 80%. While smoking, vigorous sports, education, BMI, and osteoarthritis appear to be playing a role in the prediction, the lack of moderate sports appears to be the most important risk factors as determined by the colour (red) and the length of the bar allocated to each risk factor. In contrast, as Figure 5B shows, age and the fact that the person engages in moderate sports appear to have significant impact on the prediction, which resulted in a relatively low risk of probability of 6%. Similarly, age and moderate sports appear to have a significant impact on the prediction of probabilities in both Figures 5C,D. However, while moderate sports appear to be protective for the individual as shown in Figure 5C, the relatively older age (80 years) and the lack of education appear to be the risk factors that have a significant impact on the prediction resulting in the probability of 66% of AD. In contrast, the individual shown in Figure 5D is relatively young and engages in moderate as well as vigorous sports, which appear to be the proactive factors driving the prediction with a relatively low probability of 4% risk of AD.

**Figure 6:**
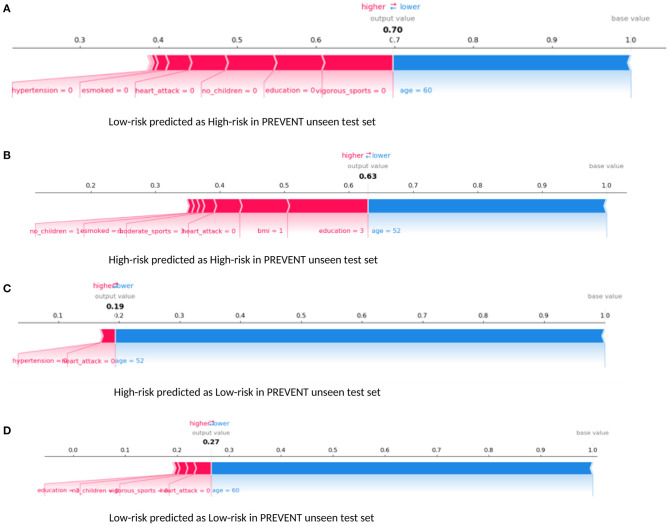
Force plot showing the effect of SHAP values on the interaction of features and the overall prediction at the individual level. This shows examples of prediction outputs PREVENT target models. Features in red show risk factors pushing up the overall probability while those in blue are protective factors pushing down the probability. **(A)** Shows PREVENT participant assigned to the Low-risk group but has been predicted by the model to be High-risk with 70% probability. **(B)** Shows a High-risk participant predicted as High-risk with 63% probability. **(C)** Shows a High-risk participant predicted as Low-risk with 19% probability. **(D)** Shows Low-risk participant predicted as Low-risk with 27% probability. Feature labels are: **esmoked** (0 = never smoked); **emotional disorders** (0 = no); **hypertension** (0 = no); **osteroarthritis** (1 = yes); **high cholesterol** (0 = no); **heart attack** (0 = no); **education** (2 = lower secondary education or second stage of basic education; 3 = upper secondary education); **moderate sports** (0 = hardly ever, or never, 1 = one to three times a month); **vigorous sports** (0 = hardly ever, or never, 1 = one to three times a month); **no children** (0 = no children); **widowed** (1 = yes); **BMI** [1 = under weight (<18.5)]; **married, living with spouse** (0 = no) and **gender** (1 = male).

Examining our target model at the individual level, Figure 6 shows randomly selected outputs when PREVENT_target model was applied to PREVENT unseen test set. Figure 6A shows a low-risk individual predicted as a high-risk with the probability of 70%. Figure 6B shows a high-risk individual correctly predicted with the probability of 7%. Figure 6C shows a high-risk individual predicted as low-risk with the probability of 19%. Figure 6D is also a low-risk individual correctly predicted as low-risk with the probability of 27%. As the figures show, while age appears to be the most protective factor for all the individuals, the lack of vigorous sports, relatively low education, and BMI appear to be the risk factors with the greatest impact. A closer look at Figure 6A shows a 60-year-old individual who has no education and lacks physical activity and therefore predicted by the model to be at high risk despite having been allocated to the low-risk group. Similarly, Figure 6B shows a 52-year-old individual belonging to the high-risk group and correctly predicted by the model with a probability of 63%. In this figure, individual age is the most protective factor, while education (3 = upper secondary level) and having a healthy weight (BMI = 1) appear to be risk factors. This may suggest that higher education may be critical for individuals with an APOE e4 gene and a parental history of dementia, compared to individuals without that fall outside the high-risk group.

## Discussion

This study developed an ensemble-based machine-learning model to predict Alzheimer's dementia risk at both population and individual levels based on the data drawn from two populations with different characteristics. Our models were built using large heterogeneous data drawn from a population of 20 European countries with up to 14 years of follow-up data. Our best model achieves high-performance accuracy, obtaining an AUROC score of 96% on the unseen test set. The decision boundaries of the best model were further updated through transfer learning. The update was done using data from a different population with different dementia risk profiles to produce a target model. The target model achieves an AUROC score of 63% and a transfer learning efficacy rate of 20%. It is also able to visualise the risk as well as protective factors that are responsible for the prediction at both population and individual levels.

To the best of our knowledge, this is the first approach that employs transfer learning with ensembles to develop dementia risk prediction models and visualisation of risk factors from an undiagnosed population in mid-life. Although numerous computational approaches have been developed, these methods have been limited in terms of sample size and the over-reliance on a homogenous sample for validation (Goerdten et al., 2019). van Maurik et al. (2019) attempted to address this issue by combining data from older adults in different populations across Europe and North America to develop dementia-risk prediction models for people with mild cognitive impairment. They employed traditional statistical modelling approaches and biomarkers, such as cerebrospinal fluid and imaging data to develop the prediction models. While we are unable to compare our proposed approach to that of van Maurik et al. (2019) due to differences in data used, it would be interesting to compare the performance of the two modelling approaches on the same dataset in the future.

Even though the relative differences in feature rankings between the models may be hard to interpret relative to their importance in predicting the dementia risk, and given that XGBoost outperforms RF as our significant test suggests, it would be reasonable to conclude that the feature rankings of XGBoost model could be more accurate and therefore reliable. The prediction models developed here identified risk factors that agree with previous literature. We demonstrate this by examining the top 10 features as ranked by the XGboost prediction models. Numerous studies have concluded that age remains the single biggest risk factor (Song et al., 2014). This is consistent with our model, ranking age to be the most important risk factor. Even though age is considered a non-modifiable risk factor, the Lancet commission report on dementia prevention by Livingston et al. (2020) identified a number of risk factors which when modified could reduce the risk of dementia by 40%. The report identified less education, hypertension, hearing impairment, smoking, obesity, depression, physical inactivity, diabetes, and infrequent social contact as potentially modifiable risk factors. Seventy percent of these risk factors were ranked among the top 10 by the study's prediction model as shown in Figure 4.

Furthermore, the interaction effects identified by the study's models are also in accordance with the existing evidence. For example, low education level is known to account for up to 8% and physical inactivity accounts for up to 3% of the dementia risk (Livingston et al., 2017). Again, both education and physical activity are associated with cognitive reserves and improvement in mental functions, suggesting that these could act as protective factors (Sharp and Gatz, 2011). Therefore, poorly educated individuals with a sedentary lifestyle could have an increased risk of dementia. This phenomenon is consistent with what is observed in Figures 5, 6. As Figure 5A demonstrates, the relatively low education and low levels of physical activity (moderate/vigorous sports) were the two major risk factors among the (non-age) other risk factors that increased the risk of dementia up 80% of this individual. This is consistent with what is observed in Figure 6A which shows an individual considered to be at low risk but due to lack of education and physical activity, the risk profile of this individual is predicted with 70% probability, with age being the only protective factor.

While the majority of the top 10 risk factors ranked by the study's prediction model were part of those identified by the recent Lancet Commission report, there are a few that appear to be playing a major role in the risk prediction but not currently part of the report. Figure 6B demonstrates the effect of emotional disorder on the risk of dementia at the individual level. Again, while age and physical activity remain significant protective factors, emotional disorder appears to be playing a significant role in the 7% risk of Alzheimer's Dementia for this individual. Therefore, any intervention in the emotional health of this participant chosen for illustrative purposes could further reduce their risk. This approach is exactly what is envisaged in the Brain Health Clinics being developed across Europe (Frisoni et al., 2020) based on a consensus led by our group in how to change clinical services for dementia prevention (Ritchie et al., 2017). This is based on collecting data from these Brain Health Clinics to support Real World machine learning approaches and using these algorithms to support the development of personalised prevention plans driven by early disease detection and comprehensive risk profiling.

Even though the performance of the study's prediction model demonstrates a potential clinical utility, we do acknowledge that it would benefit from further development and validation. Firstly, it would be beneficial to evaluate the effect of additional data sources derived from biological samples and neuroimaging on the overall performance of the study's model as well as the effect of the interactions of additional features at both population and individual levels. Secondly, further validation of the model using data from non-research settings is crucial. The dataset used in training the model is obtained from research settings, which is considered to be of high quality due to the strict data collection protocols that are used in these settings. Thirdly, the problem of imbalanced data and the ability to develop accurate prediction models that account for these problems are major challenges (Khalilia et al., 2011). However, RF and XGBoost have consistently been shown to have the capacity to handle imbalanced challenges due to the strategy employed in learning. For example, Facal et al. (2019) compared the performance of number learning algorithms, including RF and XGBoost, to predict mild cognitive impairment to dementia conversion with highly skewed class distribution, and XGBoost demonstrated superior performance over the rest of the algorithms and outperforming RF, which is consistent with the study's findings. Nevertheless, the study's model may benefit from incorporating some of the numerous imbalanced data techniques discussed by Fernández et al. (2018) in the processing pipeline as part of future work. Lastly, all missing data were removed from the training set as part of the pre-processing step, which may have led to loss of data. This approach is not ideal and sub-optimal particularly when dealing with longitudinal datasets with long follow-up periods as well as real-world datasets, which mostly have a high prevalence of missing data. Therefore, approaches to handling missing data such as those described by Buck (1960) could potentially be explored.

Even though the study's source model achieved a relatively good performance, the performance of our target model could be better. The 63% AUROC score and a transfer learning efficacy rate of 20% achieved by the study's target model could be attributed to the limited sample used to update the decision boundaries of the study's source model. This could be considered a limitation, and therefore a bigger sample size will be required to further update and evaluate the model.

## Conclusion

Drawing on the transfer learning paradigm of artificial intelligence, we developed ensemble-based models capable of predicting Alzheimer's dementia onset in a relatively younger population up to 14 years in advance of the mean in the training set with promising results. The models not only predict dementia risk but also provide a visualisation of the interactions between risk factors to determine those driving the risk prediction at the individual level. The complex nature of dementia requires powerful machine learning models to be able to learn complex patterns from the interactions between risk factors, and the study's proposed model achieves this with reasonable accuracy. While some of the risk factors identified are well-documented, our model further identified less suspected risk factors that appear to be significant in driving the risk of AD. We believe that with further development and validation, our prediction model has the potential to support the early detection for appropriate interventions to be developed to prevent dementia.

## Data Availability Statement

Publicly available datasets were analysed in this study. This data can be found at: DOIs: 10.6103/SHARE.w1.710, 10.6103/SHARE.w2.710, 10.6103/SHARE.w3.710, 10.6103/SHARE.w4.710, 10.6103/SHARE.w5.710, 10.6103/SHARE.w6.710, 10.6103/SHARE.w7.710. PREVENT WL210v1.

## Ethics Statement

The studies involving human participants were reviewed and approved by PREVENT Dementia Programme Consortium. The patients/participants provided their written informed consent to participate in this study.

## Author Contributions

SD designed the study. SD and ZZ carried out the experiments and analysed the results. All authors contributed to drafting the manuscript.

## Conflict of Interest

The authors declare that the research was conducted in the absence of any commercial or financial relationships that could be construed as a potential conflict of interest.
